# The complete chloroplast genome of a *Woodwardia japonica*

**DOI:** 10.1080/23802359.2019.1698366

**Published:** 2019-12-11

**Authors:** Rahul Vasudeo Ramekar, Ik-Young Choi, Eun Ju Cheong, Myounghai Kwak, Kyong-Cheul Park

**Affiliations:** a Department of Agriculture and Life Industry, Kangwon National University, Chuncheon, South Korea;; b Division of Forest Science, Kangwon National University, Chuncheon, South Korea;; c Plant Resources Division, National Institute of Biological Resources, Incheon, South Korea

**Keywords:** Chloroplast genome, *Woodwardia japonica*

## Abstract

*Woodwardia japonica* is one of the diverse members of the fern group and medicinally important genus. In Korea, the natural resources of *W. japonica* are being exhausted by excessive exploitation and require urgent conservation. In this study, the complete chloroplast genome of *W. japonica* was generated, and its structure was compared with that of other members of same family. The chloroplast genome was 153224 bp long, with a typical quadripartite structure including a pair of inverted repeat regions (24591 bp) separated by a large (82480 bp) and small (21562 bp) single-copy (SC) region. The genome encodes a total of 88 protein-coding genes, 35 tRNA genes, and eight rRNA genes. Additionally we identified 87 RNA editing sites in 52 genes; most of the substitution was U to C (50 sites), while C to U conversion occurred in 37 positions. The phylogenetic analysis strongly supported the relationship of *W. japonica* with *W. unigemmata* and. *A. melanocaulon* (Blechnoideae).

## Introduction

*Woodwardia japonica* (Blechnaceae) is a fern member and the most highly variable species belonging to genus *Woodwardioideae,* mainly found in well-drained slopes with deep to bright shade. The main center of diversity for *W. japonica* is located in eastern Asia, primarily in the region of Japan and Korea, Vietnam, China, and Thailand. The rhizome of *W. japonica* is believed to be effective to cure flue and verminosis and often use in traditional medicines (Liu and Gao 2011).

Here, we report on the complete chloroplast (cp) genome sequence of *W. japonica*. Plant material was collected from its natural habitat in Jejudo Is., South Korea (Voucher number: NIBRVP0000524323).

Total genomic DNA was extracted from fresh leaf tissue using a DNeasy Plant mini kit (Qiagen, Hilden, Germany). Whole genome sequencing was generated using an Illumina HiSeq 4000 platform. A total of 4.1 gb 150 bp pair-end raw reads were retrieved and quality trimmed using Trimmomatic (Bolger et al. 2014). The resultant 3.7 GB reads were then used for *de novo* assembly by the Newbler assembler (v2.9). The representative cp contigs were extracted, ordered and merged into a single draft. The cp genomes of *Woodwardia unigemmata* (KT599101.1) (Lu et al. 2015) was used as initial reference. The initial annotation of the cp genome was conducted using the DOGMA program (Wyman et al. 2004) and ARTEMIS software (Rutherford et al. 2000). In addition, all tRNA genes were verified by tRNAscan v1.21 (Schattner et al. 2005) and GC content was analyzed by MEGA 5.05 (Tamura et al. 2013). We have submitted the assembled and annotated sequence to GenBank under accession number MN587871.

**Figure 1.**
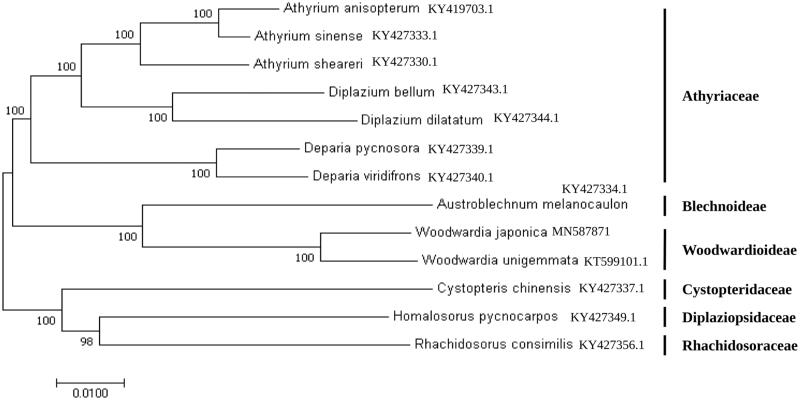
Molecular phylogenetic tree of the *Aspleniineae suborder* of fern based on the complete cp genome of 13 species.

To ascertain the phylogenetic status of *Woodwardia japonica*, the complete cp genome of 12 species belonging Aspleniineae suborder of fern family was selected. A neighbor-joining (NJ) tree was constructed with Mega 6.0 using 1000 bootstrap replicates (Tamura et al. 2013) clustered the fern species into three groups (Figure 1). All the members of family Athyriaceae (*Athyrium anisopterum*, *Athyrium sinense, Athyrium sheareri, Diplazium bellum, Diplazium dilatatum, Deparia pycnosora, Deparia viridifrons*) were clustered in one group. Another group comprised members from Woodwardioideae (*Woodwardia unigemmata* and *Woodwardia japonica*) and Blechnoideae (*Austroblechnum melanocaulon*). Members from Cystopteridaceae (*Cystopteris chinensis*), Diplaziopsidaceae (*Rhachidosorus consimilis*), Rhachidosoraceae (*Homalosorus pycnocarpos*) were placed in distinct cluster. *Woodwardia japonica, Woodwardia unigemmata* along with *Austroblechnum melanocaulon* formed a monophyletic clade with a high bootstrap value, indicating a close relationship among these species.
